# Dr. James A. Lydston

**Published:** 1891-03

**Authors:** 


Dr. James A. Lydston, late Chief of Eye and Ear Department, Washington, D. C., and Professor of chemistry in the Chicago of Physicians and Surgeons, has removed to Denver, Colo., where he will re-enter the practice of his specialty.

The change of location has been necessitated by the illness of his wife.

